# Testosterone replacement therapy in men who conceived with intracytoplasmic sperm injection: nationwide register study

**DOI:** 10.1530/EJE-19-0734

**Published:** 2020-02-11

**Authors:** Angel Elenkov, Yahia Al-Jebari, Yvonne Lundberg Giwercman, Aleksander Giwercman

**Affiliations:** 1 Lund University, Molecular Genetic Reproductive Medicine, Malmö, Sweden; 2 Skåne University Hospital Malmö, Reproductive Medicine Center, Malmö, Sweden

## Abstract

**Objectives:**

Male hypogonadism is associated with higher risk of co-morbidity and premature mortality. It is, therefore, of utmost importance to identify young men who are at the highest risk of testosterone deficiency and who may benefit from preventive measures. In this context, infertile men constitute a high-risk group. The extent of testosterone replacement therapy (TRT) among infertile men, defined as men who have to undergo assisted reproduction for fatherhood, is currently unknown. Therefore, we evaluated the pattern of prescription of TRT in the years following child conception among men who have fathered children with the help of intracytoplasmic sperm injection (ICSI) or *in vitro* fertilization (IVF).

**Design:**

By sourcing data from national population registries, hazard ratio (HR) for subsequent TRT was assessed for IVF and ICSI-treated men and compared to those who conceived spontaneously with age Cox regression analysis adjusted for age, educational level and previous intake of medicines for metabolic diseases.

**Results:**

ICSI and IVF fathers had increased incidence of newly prescribed TRT compared to fathers conceiving spontaneously (ICSI: HR = 3.81, 95% CI = 3.09–4.69, *P* < 0.001; IVF: HR = 1.54, 95% CI = 1.15–2.05, *P* = 0.003). After adjustment for prescription of medication for one or more components of the MetS prior to TRT, the risk estimates attenuated but remained robust both for ICSI-treated (HR = 3.17 (95% CI: 2.56–3.9) and IVF-treated men (HR = 1.06 (95% CI: 1.05–1.07).

**Conclusion:**

Men who have to utilise powerful techniques, such as ICSI for fathering children, may be at risk for testosterone deficiency. Routine endocrine evaluation of men seeking fertility treatment is hence warranted.

## Introduction

Low serum testosterone in men (hypogonadism) has been associated with higher mortality and increased co-morbidity including cardiovascular disease, diabetes metabolic syndrome (1, 2, 3), low grade inflammation (4), lower insulin sensitivity (5) and accelerated atherosclerosis (6). In addition, hypogonadism has been associated with higher mortality (7).

On the other hand, chronic diseases and obesity may also lead to lower testosterone levels (8) and thus complicate the establishment of a causal relationship with testosterone deficiency. However, patients on androgen deprivation therapy for prostate cancer are at an increased risk of coronary heart disease, diabetes and cardiovascular death (1), indicating a key role for testosterone in atheroprotection.

Nevertheless, in the last decades, increasing rates of testosterone prescriptions worldwide have risen concerns of possible overtreatment (9, 10), possibly due to contradicting results regarding the safety and efficacy of testosterone treatment. The number of studies, in particular randomised controlled studies, is however limited, and inclusion of elderly men with higher co-morbidity and often short follow-up result in available data being rather inconclusive (11, 12). It is therefore of utmost importance to identify young men who are at the highest risk of testosterone deficiency and who may benefit from preventive measures.

In this context, infertile men constitute a high-risk group. Previous studies have shown that up to 30% of men with fertility issues and sperm concentration below 20 × 10^6^/mL are presenting with biochemical signs of hypogonadism, that is, low serum testosterone and/or high luteinizing hormone (LH) (13). Men with low sperm counts (<39 million/ejaculate) had up to 12-fold increased risk for hypogonadism (14) and hence could be regarded as a high-risk group for testosterone deficiency and subsequent co-morbidity. However, since exogenous testosterone suppresses gonadotropin secretion and thereby spermatogenesis, prescription of testosterone replacement is contraindicated in men wishing to become fathers. Still, one should expect that, if properly examined, men with poor semen quality and infertility problems should receive testosterone replacement therapy (TRT) more often after a successful fertility treatment, that is, intra cytoplasmic sperm injection (ICSI) or conventional *in vitro* fertilization (IVF).

In Sweden, ICSI treatment is mainly used in cases with significantly impaired semen quality as the major indication for ICSI (15), whereas conventional IVF, in which sperms are allowed to fertilize retrieved oocytes in a laboratory dish, is the standard procedure. Therefore, in a previous research, ICSI treatment has been used as a feasible proxy for identifying men with impaired fertility in population based registries (16, 17).

Our aim was, by using the nationwide register data, to evaluate the pattern of prescription of TRT in the years following child conception among men who have fathered children with the help of ICSI or standard IVF.

## Subjects and methods

### Study population and data acquisition

Data used in the current study were sourced from national registries. From the Swedish Medical Birth Register (SMBR), Total Population Register and the Multigenerational Register, data on all fathers to children born alive during the period between January 1, 2006 and December 31, 2016 were retrieved. SMBR contains information on 97–99% of all births in Sweden from 1973 onward (10). By cross-linking the identity codes of the mothers to the Swedish Total Population Register and the Swedish Multigenerational Register, the fathers were identified. As SMBR contains data on the birth date and the gestational week of the newborn, the approximate time of conception could be calculated. Subsequently, the data on mothers and fathers were matched with the Swedish National Quality Register for Assisted Reproduction, which contains information on assisted reproduction method (ICSI or IVF) for all births in Sweden from 2007 and onward. Conception methods before 2007 were gathered from the SMBR.

**Figure 1:**
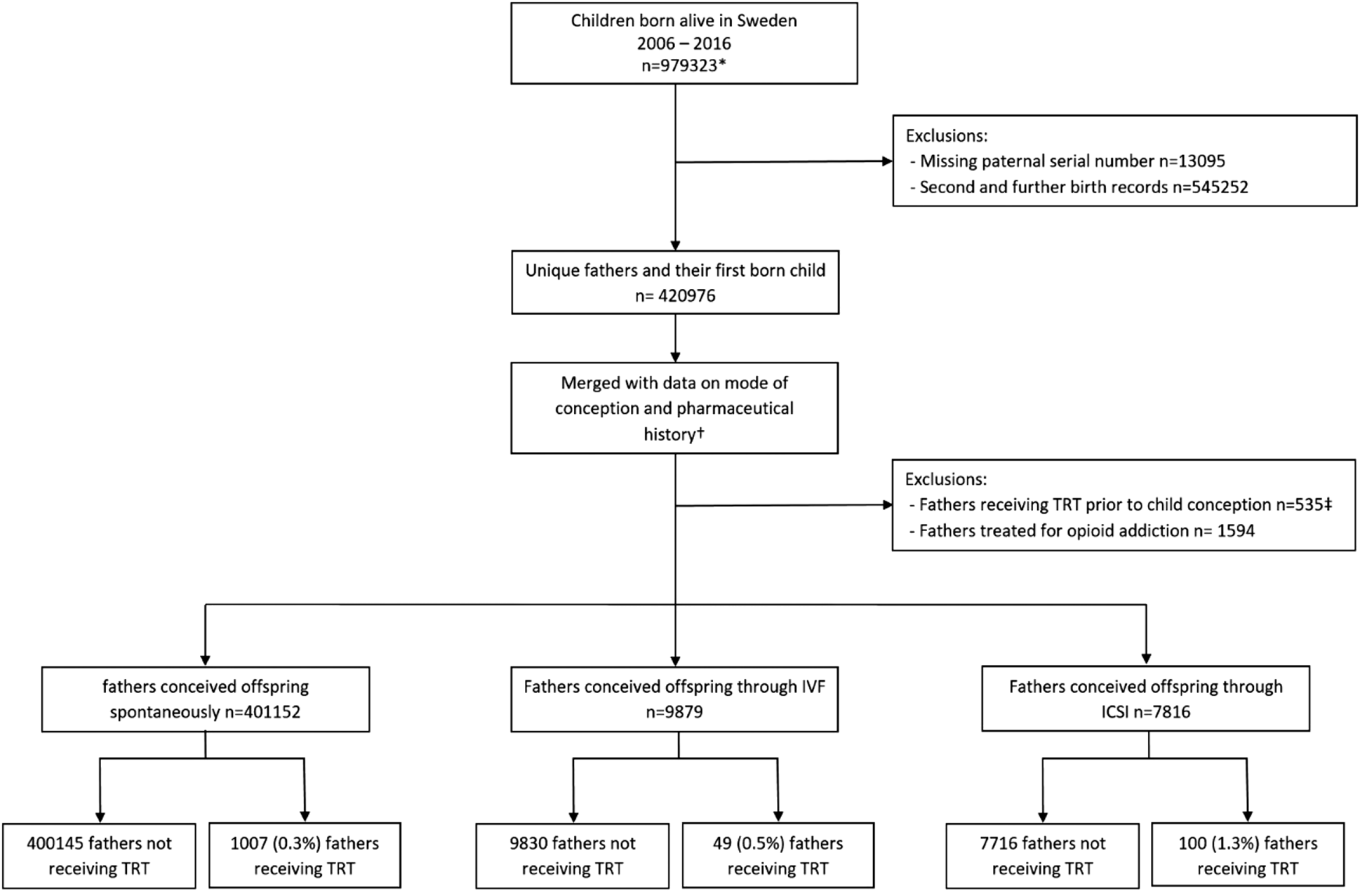
Data acquisition using Swedish nationwide registries. TRT: Testosterone replacement therapy; IVF: *in vitro* fertilisation; ICSI: intracytoplasmic sperm injection.

The men were categorized according to the mode of conception, ICSI, IVF or natural conception (control group) for the first child registered in SMBR (*n* = 420 976, Fig. 1). Pharmaceutical history including TRT was sourced from the Swedish Prescription Drug Register (SPDR), estimated to cover more than 99% of all prescriptions in Sweden (18). Reporting of fertility treatments to the national registers is mandated by law for both private and public clinics with expected completeness of up to 100% (19).

The study was approved by the Regional Ethical committee in Lund (No 2015/670).

### Statistical analysis

Hazard ratios (HR) and 95% confidence intervals (CI) for TRT were calculated using the Cox proportional hazards model with accompanying Kaplan–Meier curves. Timescale was defined as the time on study with men entering at time of conception, adjusting for age, as continuous co-variate, educational level (categorical: ≤10, 11–14, ≥15 years). Cases of missing paternal serial number and missing conception estimate for child (because of missing gestational age at birth) were regarded as missing data and were hence excluded. To avoid bias introduced by fathers being counted multiple times, only the conception method for the first child born was used for categorizing the fathers.

Since TRT is known to have a negative effect on sperm production, fathers who had been prescribed to testosterone treatment 6 months before estimated child conception date were excluded from further analysis (535 men). Moreover, as long-term treatment for opioid addiction is known to hamper the hypothalamo-gonadal axis, resulting in reduced serum testosterone level, also this group of men was excluded from the analysis (1594 men), leaving in total 418 847 men in the study.

Associated co-morbidities, especially metabolic, are known to be associated with low T levels (8). By using the SPDR, we looked at the pattern of filled drug prescriptions for one or more components of the metabolic syndrome (MetS) – diabetes mellitus (DM), hypertension (HT) and dyslipidemia (DLE). Corresponding generic medications of at least one prescription for each disease were used as proxy to identify men treated for DM, HT or DLE among the study groups as described previously (17). We then identified those who were prescribed to these medicines before the TRT. These variables (’history of medicine for AD, AHT or DLE’ prior to TRT – yes/no) were included in a separate regression model with the previously mentioned adjustment in order to explore the effect of metabolic disturbances on the risks of receiving TRT.

Since non-prescription of TRT, despite low testosterone levels and symptoms of hypogonadism, could be due to a wish of having more children subsequent to the birth of the index child, we also estimated the frequency of subsequent childbirth by using descriptive statistics. In the latter analysis, only fathers who have conceived their children in the period between January 1, 2006 and December 31, 2009 were included, in order to allow at least 7 years of follow-up time.

## Results

### Background characteristics

Of the total cohort of 418 847 men, 17 695 (4%) conceived by assisted reproduction: 7816 men by ICSI and 9879 with IVF. Incident prescription rates of TRT were 1.3% among the ICSI-treated men, 0.5% in the IVF group and 0.3% among the controls. Mean (s.d.) age at time of conception was 36.3 years (6.1) for ICSI-treated men, 35.8 years (5.4) for IVF fathers and 31.6 years (6.3) for the controls, respectively. Data on educational level are presented in Table 1.

**Table 1:** Educational level in years among the study groups.

	Educational level (*n*/%)			
	≤10 years	11–14 years	≥15 years	Total
Fathers treated with ICSI	572 (7.3%)	3871 (49%)	3452 (44%)	7816
Fathers treated with IVF	556 (5.6%)	4604 (46.6%)	4741 (47.9%)	9879
Fathers who conceived spontaneously	46 484 (11.6%)	204 307 (50.9%)	145 810 (36.3%)	401 152

Of the 2352 fathers who had their first child conceived with ICSI during the period between 2006 and 2009, 1842 (78.3%) had a second child. Of those men, 40 (2.2%) became fathers with IVF treatment, 1036 (56.2%) with ICSI and 766 (41.6%) spontaneously. Of the 3401 fathers who had their first child born after IVF, 2633 (77.4%) had a second child, 944 (35.9%) with IVF treatment, 138 (5.2%) after ICSI and 1551 (58.9) spontaneously. From the control group, consisting of 162 050 fathers, 134 493 (83.0%) had a second child. Of these men, 558 (0.4%) underwent IVF, 392 (0.3%) ICSI and 133 543 (99.7%) conceived spontaneously.

### Incident TRT prescription

Median time between child conception and TRT prescription was 4.41 years. At the fifth year of follow-up, TRT was prescribed to 0.82% of ICSI-treated men (38/4647), 0.28% (16/5612) of the IVF fathers and 0.13% (398/287 253) of the controls. At the tenth year of follow-up, the TRT prescription rates were 2.81% (21/746), 0.84% (9/1066) and 0.39% (154/39 221), respectively. Among the men who received TRT, 14% were receiving a therapy for HT (163/1156), 3.3% for AD (39/1156) and 5.6% for DLE (65/1156).

**Figure 2:**
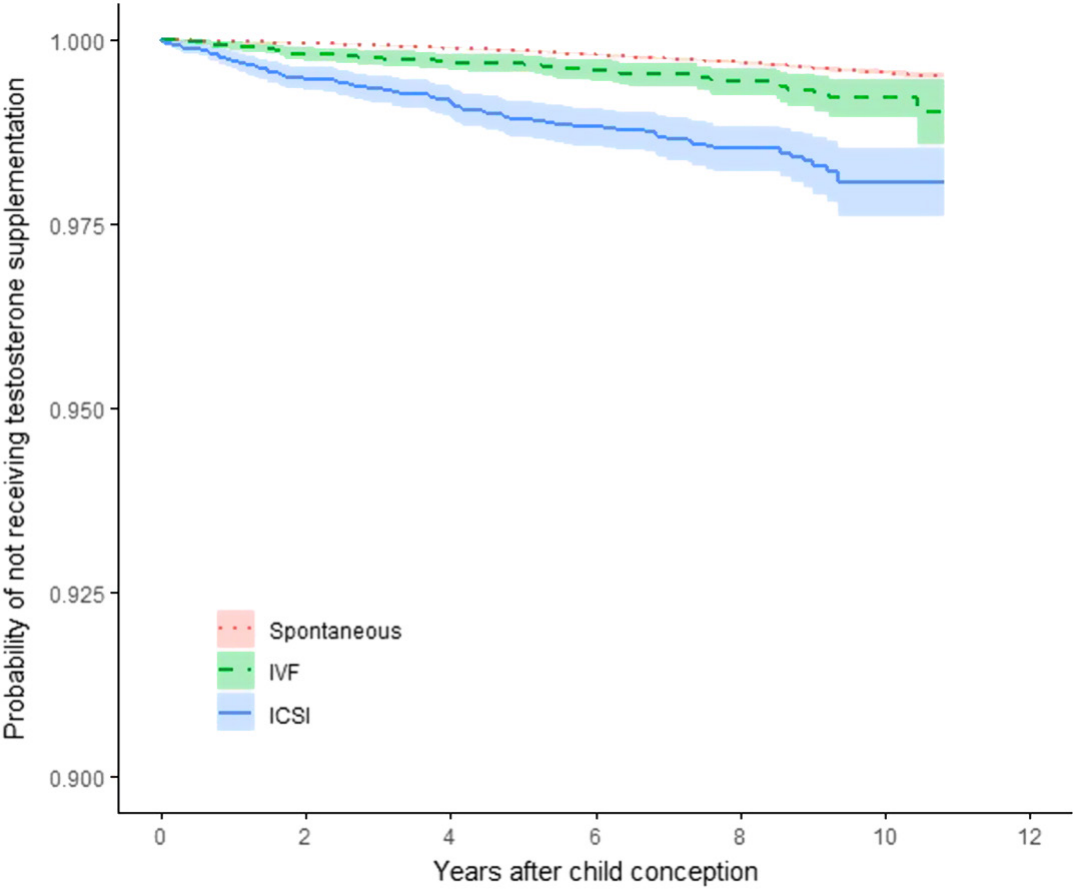
Kaplan–Meier survival curve of testosterone supplementation for ICSI- and IVF-fathers, respectively, as compared to spontaneously conceiving fathers. Shaded area denotes 95% confidence intervals. Log rank *P* value <0.001. Topmost line indicates fathers conceiving spontaneously, middle line IVF and bottom line ICSI.

After a mean follow-up period of 6.7 years, age and educational level adjusted HRs of TRT prescriptions were statistically significantly increased in ICSI and IVF fathers as compared to controls (HR = 3.81, 95% CI = 3.09–4.69, *P* < 0.001 in the ICSI and HR = 1.54, 95% CI = 1.15–2.05, *P* = 0.003 in the IVF group, respectively; Fig. 2). The ICSI group also received more prescriptions when compared to IVF men (HR = 2.54, CI = 1.80–3.58, *P* < 0.001).

After additional adjustment for prescription of medication for one or more components of the MetS prior to TRT, the risk estimates attenuated but remained robust both for ICSI-treated (HR = 3.17 (95% CI: 2.56–3.9) and IVF-treated men (HR = 1.06 (95% CI: 1.05–1.07).

## Discussion

Men who conceived by means of assisted reproduction had significantly higher incident prescription of TRT compared to men conceiving naturally, with almost four times more prescriptions in the ICSI-treated group and 1.5 times increase in the IVF-treated men, respectively. The results remained robust even after adjusting for prescription of medication for MetS. Therefore, impaired androgenic state likely is overrepresented among ICSI-treated men.

However, although low testosterone levels are known to affect life quality and health negatively, TRT was prescribed to a relatively limited proportion of men. The common indication for initiating androgen replacement is a combination of low testosterone levels and symptoms of hypogonadism including sexual dysfunction (20). In the same cohort of sub-fertile men, in which 30% prevalence of biochemical signs of androgen deficiency was found, the odds ratio of symptoms of impaired sexual function was two to three times increased as compared to age-matched controls (21). Since an endocrine evaluation is currently not a part of routine investigation of men from infertile couples, the proportions of TRT incidence of up to 2.8%, depending on the length of follow-up, may represent an underestimation of the actual TRT necessity. This apparent under-treatment can either be related to under-diagnosis or to reluctance in prescription of TRT, in general, and to men who may want to father more children in particular. The latter argument was supported by the fact that almost 80% of all ICSI fathers had a second child.

The inclusion of a large nationwide cohort with large number of subjects is a major strength of this study. In addition, the registries have a coverage of almost 100%. However, a number of limitations are present. The observational nature of the analysis precludes evaluation of causal relationships, making us unable to differentiate those who presented with a combination of low testosterone and clinical symptoms of testosterone deficiency, which is defined as the criterion for TRT (22). Furthermore, the partner’s fertility status was unknown and, in addition, men who never succeeded to become fathers were not registered. Some of these couples could have undergone insemination or IVF with donated gametes, leading to selection bias. However, this would rather dilute the results and reduce the difference between the groups than strengthen it. One could also claim that men referred for fertility treatment due to the contact with the health care system have higher chance of being diagnosed with hypogonadism. However, the difference between ICSI and IVF fathers, who also are in need of health care, indicates that the former group indeed is a high-risk group for being hypogonadal. Furthermore, the majority of TRT prescriptions was initiated many years after child conception, and the gap between ICSI fathers and those who did not undergo assisted reproduction was increasing throughout the entire follow-up period. A number of chronic diseases are known to negatively affect T levels (8). Despite adjusting for history of one or more components of MetS, the usage of prescription medicine as a proxy for disease has its shortcomings. Some patients might not receive medications for their metabolic disturbance, but rather advised about lifestyle changes. Therefore, the inability to fully adjust for co-morbidity at the time of initiation of TRT remains a major limitation. However, in a previous study, we found only 8% of men referred for infertility work-up being on hormonal – non TRT – replacement or treatment for one or more symptoms of metabolic syndrome (13).

## Conclusion

Young sub-fertile, in particular, ICSI-treated men constitute a risk group for testosterone deficiency with its inherent risks for long-term morbidity and mortality if left untreated. Hence, despite some recommendations against screening of testosterone levels in the general population (22), routine endocrine investigation of men seeking infertility treatment is warranted, necessitating the need for randomized studies regarding efficacy of TRT given to younger hypogonadal men in preventing long-term morbidity. As a significant part of men who fathered their first child by use of ICSI or IVF succeed to get more children without the use of assisted reproduction, men undergoing fertility treatment, being in contact with the health care system, represent a target group for endocrine testing. Although the TRT may be postponed in men wishing to father more children, a follow-up of these men should be undertaken.

## Declaration of interest

The authors declare that there is no conflict of interest that could be perceived as prejudicing the impartiality of this study.

## Funding

The study was supported by grants from the Interreg V funded program ReproUnion and by joint European Association of Urology – ReproUnion scholarships.

## Data access and responsibility

Y A and A E had full access to all the data in the study and take responsibility for the integrity of the data and the accuracy of the data analysis. Data cannot be shared because they are from registries or institutional databases of patients providing routinely collected data.
